# Supplementary material for: Improving deep learning-based segmentation of diatoms in gigapixel-sized virtual slides by object-based tile positioning and object integrity constraint
Source: PLoS One. 2023 Feb 24;18(2):e0272103. doi: 10.1371/journal.pone.0272103 (PMC9956069; doi:10.1371/journal.pone.0272103)
Supplement: S3 Dataset — https://doi.org/10.5281/zenodo.7079072. (DOCX) [file pone.0272103.s003.docx]

**S3 Dataset. Mask R-CNN and U-Net segmentation models and experimental results on segmenting virtual slide images of diatom preparations from river Menne.**

<https://doi.org/10.5281/zenodo.7079072>
