# Supplementary material for: Improving deep learning-based segmentation of diatoms in gigapixel-sized virtual slides by object-based tile positioning and object integrity constraint
Source: PLoS One. 2023 Feb 24;18(2):e0272103. doi: 10.1371/journal.pone.0272103 (PMC9956069; doi:10.1371/journal.pone.0272103)
Supplement: S4 Dataset — https://doi.org/10.5281/zenodo.7107456. (DOCX) [file pone.0272103.s004.docx]

**S4 Dataset. Segmentation performance evaluation results and R scripts for generating Figs 5 and 6 as well as Tables 3 and 4.**

<https://doi.org/10.5281/zenodo.7107456>
