# Supplementary material for: Improving deep learning-based segmentation of diatoms in gigapixel-sized virtual slides by object-based tile positioning and object integrity constraint
Source: PLoS One. 2023 Feb 24;18(2):e0272103. doi: 10.1371/journal.pone.0272103 (PMC9956069; doi:10.1371/journal.pone.0272103)
Supplement: S1 Fig — Investigated factors are model architecture, tiling method (FS = fixed-stride, OBP = object-based positioning, OBP+OIC = object-based positioning + object integrity constraint), training data set size (coded in the y-axis labels) and prediction threshold (coded in colors). Crosses depict mean, vertical lines within boxes median values. (PDF) [file pone.0272103.s005.pdf]

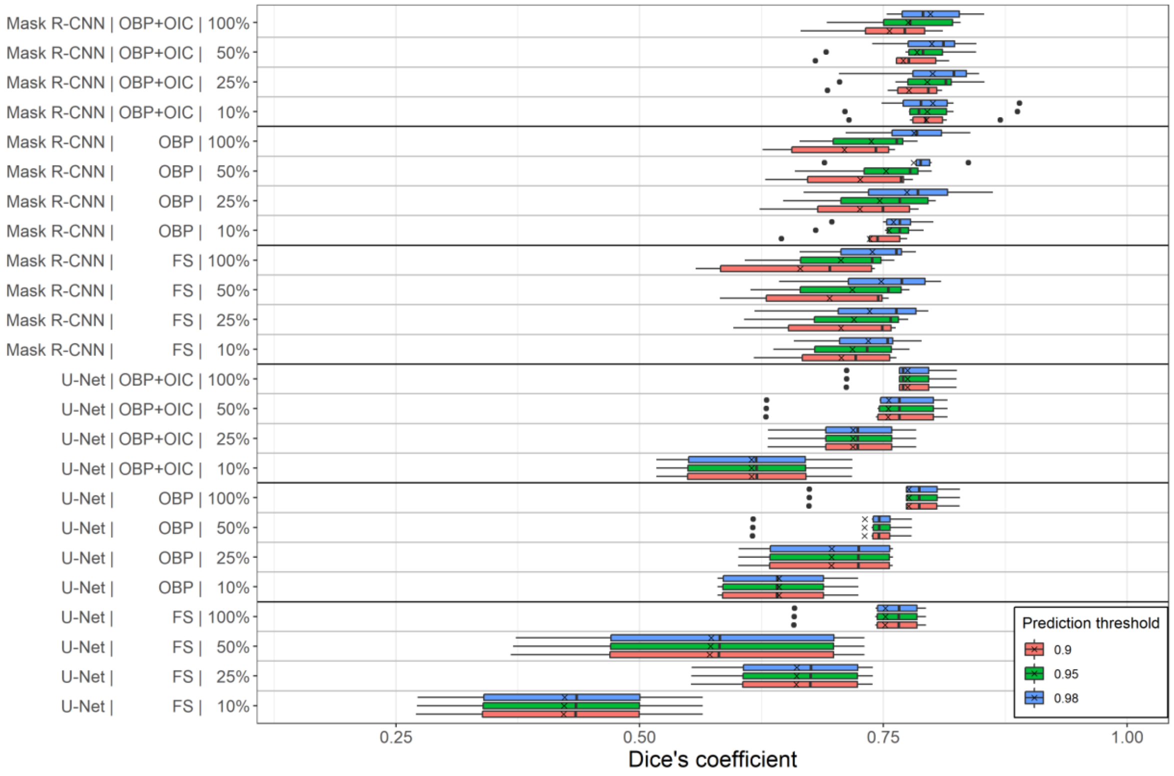
Mask R-CNN | OBP+OIC | 100%
Mask R-CNN | OBP+OIC | 50%
Mask R-CNN | OBP+OIC | 25%
Mask R-CNN | OBP+OIC | 10%
Mask R-CNN | OBP | 100%
Mask R-CNN | OBP | 50%
Mask R-CNN | OBP | 25%
Mask R-CNN | OBP | 10%
Mask R-CNN | FS | 100%
Mask R-CNN | FS | 50%
Mask R-CNN | FS | 25%
Mask R-CNN | FS | 10%
U-Net | OBP+OIC | 100%
U-Net | OBP+OIC | 50%
U-Net | OBP+OIC | 25%
U-Net | OBP+OIC | 10%
U-Net | OBP | 100%
U-Net | OBP | 50%
U-Net | OBP | 25%
U-Net | OBP | 10%
U-Net | FS | 100%
U-Net | FS | 50%
U-Net | FS | 25%
U-Net | FS | 10%
Prediction threshold
0.9
0.95
0.98
0.25
0.50
0.75
1.00
Dice's coefficient

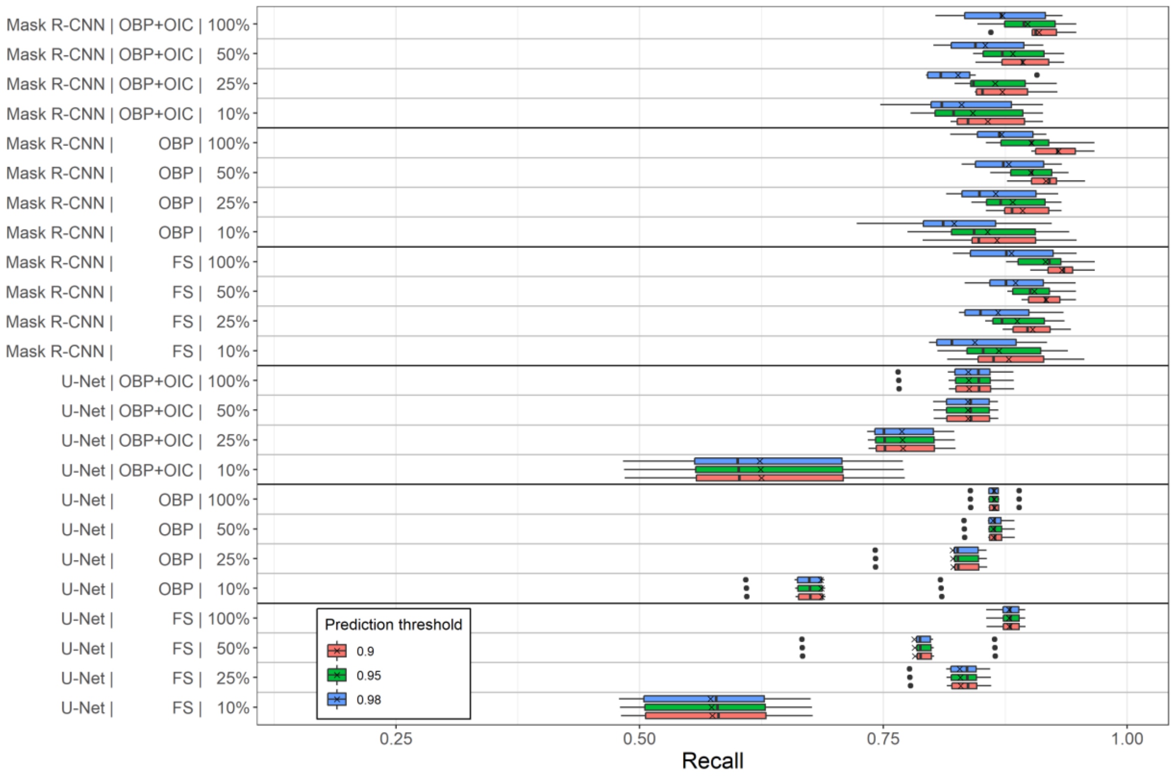
Mask R-CNN | OBP+OIC | 100%
Mask R-CNN | OBP+OIC | 50%
Mask R-CNN | OBP+OIC | 25%
Mask R-CNN | OBP+OIC | 10%
Mask R-CNN | OBP | 100%
Mask R-CNN | OBP | 50%
Mask R-CNN | OBP | 25%
Mask R-CNN | OBP | 10%
Mask R-CNN | FS | 100%
Mask R-CNN | FS | 50%
Mask R-CNN | FS | 25%
Mask R-CNN | FS | 10%
U-Net | OBP+OIC | 100%
U-Net | OBP+OIC | 50%
U-Net | OBP+OIC | 25%
U-Net | OBP+OIC | 10%
U-Net | OBP | 100%
U-Net | OBP | 50%
U-Net | OBP | 25%
U-Net | OBP | 10%
U-Net | FS | 100%
U-Net | FS | 50%
U-Net | FS | 25%
U-Net | FS | 10%
Prediction threshold
0.9
0.95
0.98
0.25
0.50
0.75
1.00
Recall

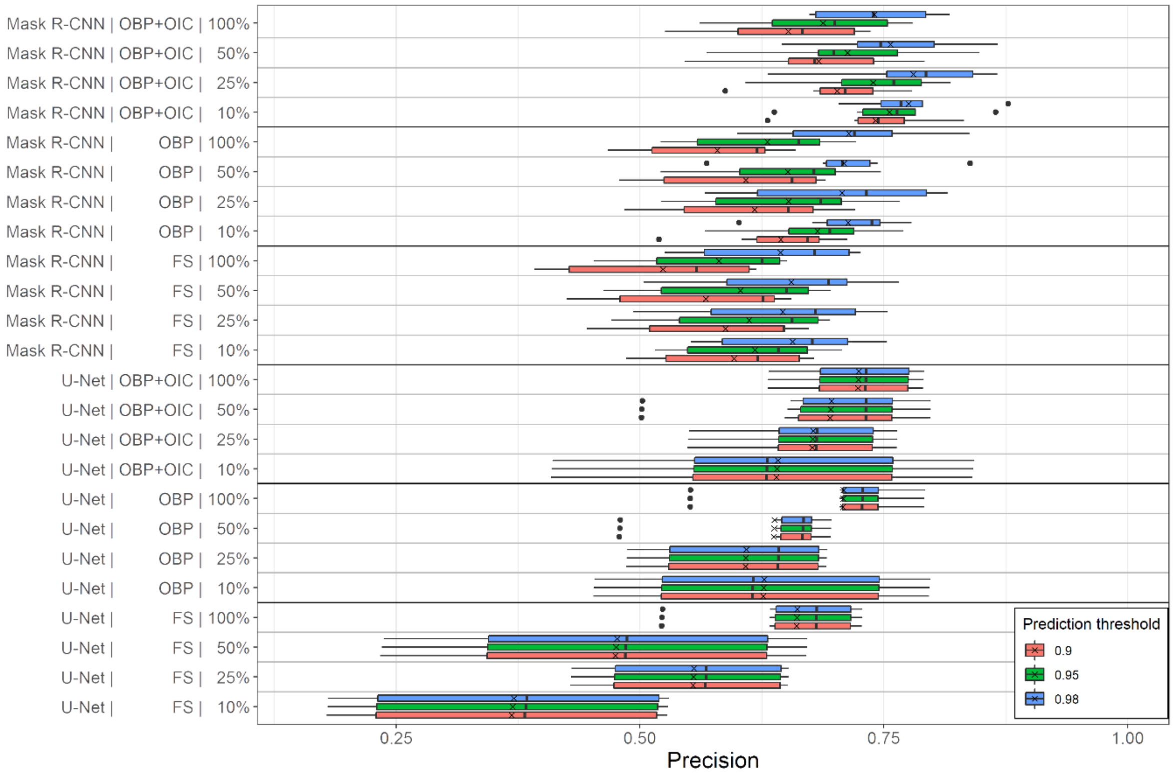

S5 Fig: Boxplots of segmentation performance scores. *Investigated factors are model architecture, tiling method (FS = fixed-stride, OBP = object-based positioning, OBP+OIC = object-based positioning + object integrity constraint), training data set size (coded in the y-axis labels) and prediction threshold (coded in colors). Crosses depict mean, vertical lines within boxes median values.*
