# Supplementary figures and images for: Improving deep learning-based segmentation of diatoms in gigapixel-sized virtual slides by object-based tile positioning and object integrity constraint
Source: PLoS One. 2023 Feb 24;18(2):e0272103. doi: 10.1371/journal.pone.0272103 (PMC9956069; doi:10.1371/journal.pone.0272103)

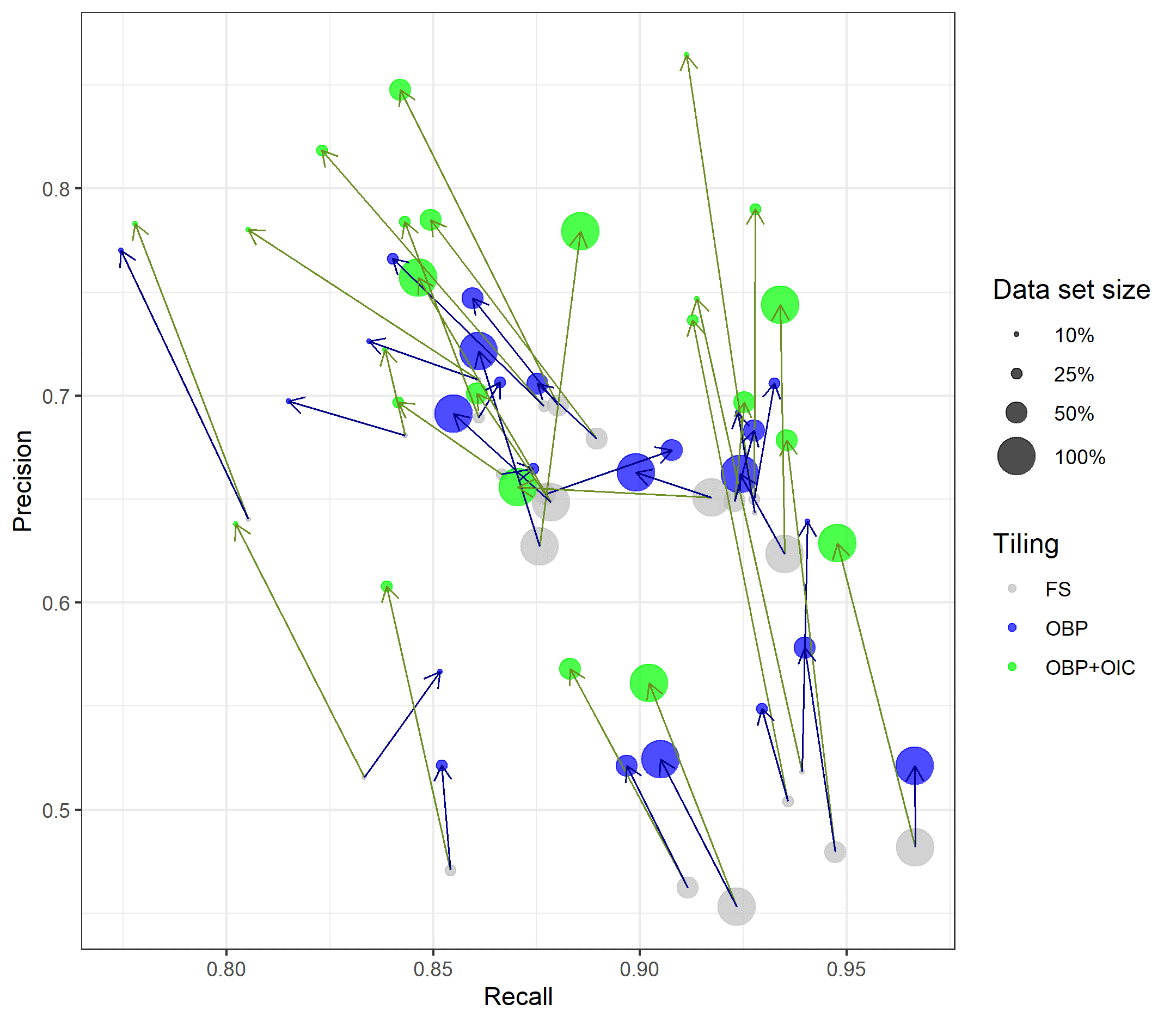

Supplement: S2 Fig — Blue arrows indicate shift from fixed-stride to object-based tile positioning, green arrows indicate shift to object-based positioning + object integrity constraint. FS = fixed-stride, OBP = object-based positioning, OBP+OIC = object-based positioning + object integrity constraint. Please note the different axis scalings, i.e., that substantial improvements in precision with the object-based tiling schemes are usually associated with relatively much smaller decreases in recall. (TIFF) [file pone.0272103.s006.tiff]

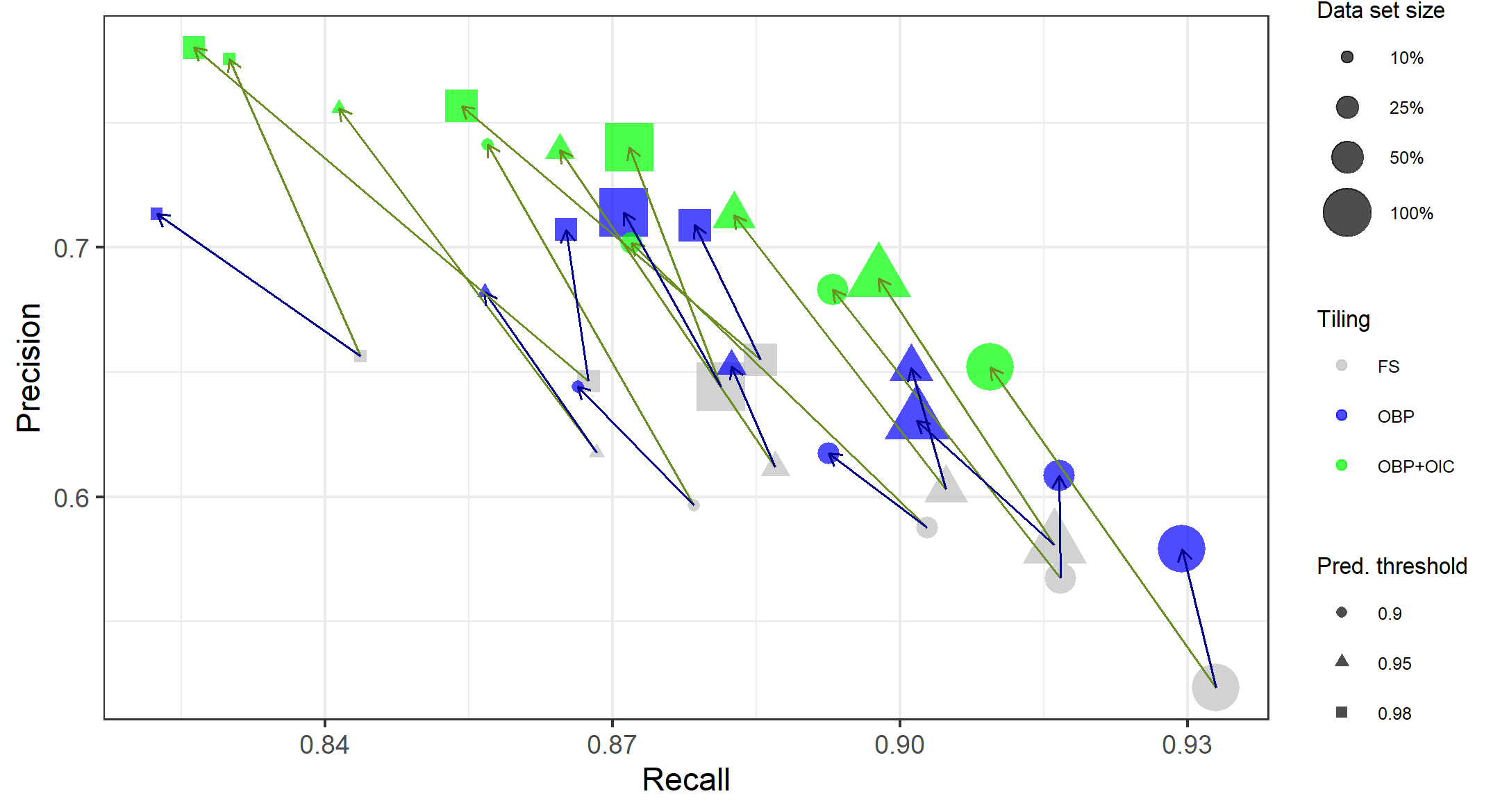

Supplement: S3 Fig — Blue arrows indicate shift from fixed-stride to object-based tile positioning, green arrows indicate shift to object-based positioning + object integrity constraint. FS = fixed-stride, OBP = object-based positioning, OBP+OIC = object-based positioning + object integrity constraint; Pred. threshold = prediction threshold. Please note the different axis scalings, i.e., that substantial improvements in precision with the object-based tiling schemes are usually associated with relatively much smaller decreases in recall. (TIFF) [file pone.0272103.s007.tiff]

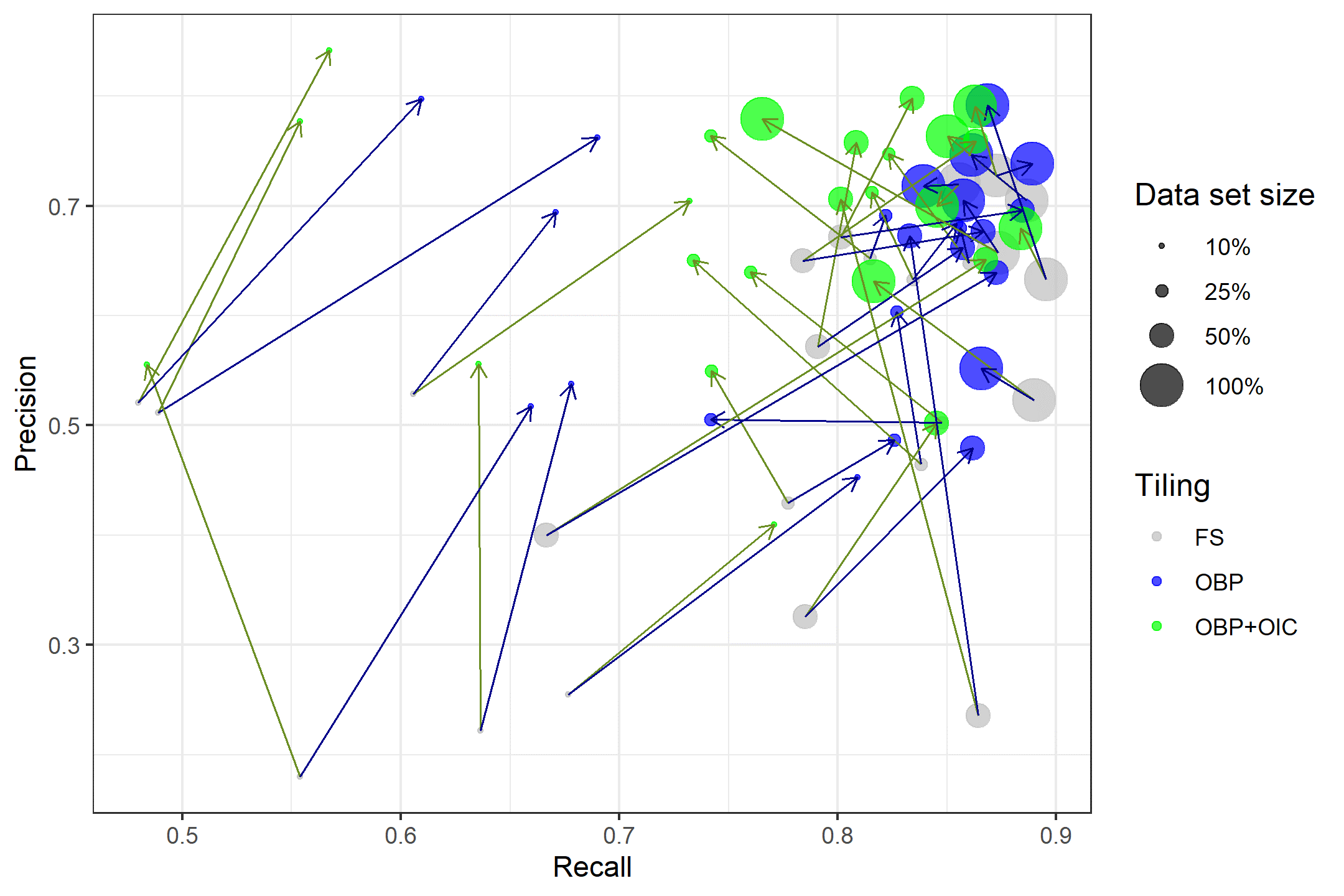

Supplement: S4 Fig — Results for prediction thresholds 0.90 and 0.98 are nearly identical. Blue arrows indicate shift from fixed-stride to object-based tile positioning, green arrows indicate shift to object-based positioning + object integrity constraint. FS = fixed-stride, OBP = object-based positioning, OBP+OIC = object-based positioning + object integrity constraint. Please note the different axis scalings, i.e., that substantial improvements in precision with the object-based tiling schemes are usually associated with relatively much smaller decreases in recall. (TIFF) [file pone.0272103.s008.tiff]
