# Supplementary material for: Improving deep learning-based segmentation of diatoms in gigapixel-sized virtual slides by object-based tile positioning and object integrity constraint
Source: PLoS One. 2023 Feb 24;18(2):e0272103. doi: 10.1371/journal.pone.0272103 (PMC9956069; doi:10.1371/journal.pone.0272103)
Supplement: S1 Table — PT = Prediction Threshold, Dice = Dice’s coefficient, FS = fixed-stride, OBP = object-based positioning, OBP+OIC = object-based positioning + object integrity constraint. (PDF) [file pone.0272103.s009.pdf]

S9 Table: Segmentation performance (means over the six virtual slides).

Architecture	Data set size	Tiling	PT	Dice	Recall	Precision
Mask R-CNN	10%	FS	0.9	0.707	0.878	0.596
Mask R-CNN	10%	FS	0.95	0.718	0.868	0.618
Mask R-CNN	10%	FS	0.98	0.734	0.844	0.656
Mask R-CNN	10%	OBP	0.9	0.736	0.866	0.644
Mask R-CNN	10%	OBP	0.95	0.756	0.857	0.682
Mask R-CNN	10%	OBP	0.98	0.760	0.822	0.713
Mask R-CNN	10%	OBP+OIC	0.9	0.794	0.857	0.741
Mask R-CNN	10%	OBP+OIC	0.95	0.795	0.841	0.756
Mask R-CNN	10%	OBP+OIC	0.98	0.800	0.830	0.775
Mask R-CNN	25%	FS	0.9	0.706	0.903	0.588
Mask R-CNN	25%	FS	0.95	0.720	0.887	0.612
Mask R-CNN	25%	FS	0.98	0.736	0.867	0.646
Mask R-CNN	25%	OBP	0.9	0.726	0.893	0.617
Mask R-CNN	25%	OBP	0.95	0.746	0.882	0.652
Mask R-CNN	25%	OBP	0.98	0.774	0.865	0.707
Mask R-CNN	25%	OBP+OIC	0.9	0.776	0.872	0.702
Mask R-CNN	25%	OBP+OIC	0.95	0.795	0.865	0.739
Mask R-CNN	25%	OBP+OIC	0.98	0.800	0.826	0.780
Mask R-CNN	50%	FS	0.9	0.695	0.917	0.567
Mask R-CNN	50%	FS	0.95	0.718	0.905	0.603
Mask R-CNN	50%	FS	0.98	0.748	0.885	0.655
Mask R-CNN	50%	OBP	0.9	0.726	0.917	0.608
Mask R-CNN	50%	OBP	0.95	0.753	0.901	0.652
Mask R-CNN	50%	OBP	0.98	0.781	0.879	0.709
Mask R-CNN	50%	OBP+OIC	0.9	0.770	0.893	0.683
Mask R-CNN	50%	OBP+OIC	0.95	0.784	0.883	0.713
Mask R-CNN	50%	OBP+OIC	0.98	0.800	0.854	0.757
Mask R-CNN	100%	FS	0.9	0.664	0.933	0.523
Mask R-CNN	100%	FS	0.95	0.706	0.916	0.581
Mask R-CNN	100%	FS	0.98	0.739	0.881	0.644
Mask R-CNN	100%	OBP	0.9	0.710	0.929	0.579
Mask R-CNN	100%	OBP	0.95	0.738	0.902	0.631
Mask R-CNN	100%	OBP	0.98	0.781	0.871	0.714
Mask R-CNN	100%	OBP+OIC	0.9	0.756	0.909	0.652
Mask R-CNN	100%	OBP+OIC	0.95	0.775	0.898	0.688
Mask R-CNN	100%	OBP+OIC	0.98	0.798	0.872	0.740
U-Net	10%	FS	0.9	0.422	0.574	0.369
U-Net	10%	FS	0.95	0.422	0.574	0.370
U-Net	10%	FS	0.98	0.423	0.572	0.371
U-Net	10%	OBP	0.9	0.643	0.687	0.626
U-Net	10%	OBP	0.95	0.643	0.686	0.627
U-Net	10%	OBP	0.98	0.643	0.686	0.627

U-Net	10%	OBP+OIC	0.9	0.615	0.625	0.640
U-Net	10%	OBP+OIC	0.95	0.615	0.624	0.641
U-Net	10%	OBP+OIC	0.98	0.615	0.623	0.641
U-Net	25%	FS	0.9	0.661	0.829	0.554
U-Net	25%	FS	0.95	0.661	0.829	0.555
U-Net	25%	FS	0.98	0.661	0.828	0.555
U-Net	25%	OBP	0.9	0.697	0.821	0.608
U-Net	25%	OBP	0.95	0.697	0.821	0.608
U-Net	25%	OBP	0.98	0.697	0.821	0.609
U-Net	25%	OBP+OIC	0.9	0.719	0.770	0.677
U-Net	25%	OBP+OIC	0.95	0.719	0.770	0.677
U-Net	25%	OBP+OIC	0.98	0.719	0.769	0.678
U-Net	50%	FS	0.9	0.572	0.782	0.475
U-Net	50%	FS	0.95	0.572	0.782	0.476
U-Net	50%	FS	0.98	0.573	0.782	0.477
U-Net	50%	OBP	0.9	0.730	0.863	0.637
U-Net	50%	OBP	0.95	0.731	0.863	0.638
U-Net	50%	OBP	0.98	0.731	0.862	0.638
U-Net	50%	OBP+OIC	0.9	0.755	0.837	0.695
U-Net	50%	OBP+OIC	0.95	0.755	0.837	0.696
U-Net	50%	OBP+OIC	0.98	0.755	0.836	0.696
U-Net	100%	FS	0.9	0.752	0.879	0.661
U-Net	100%	FS	0.95	0.752	0.879	0.661
U-Net	100%	FS	0.98	0.752	0.879	0.661
U-Net	100%	OBP	0.9	0.776	0.864	0.708
U-Net	100%	OBP	0.95	0.776	0.864	0.708
U-Net	100%	OBP	0.98	0.776	0.863	0.709
U-Net	100%	OBP+OIC	0.9	0.775	0.838	0.724
U-Net	100%	OBP+OIC	0.95	0.775	0.837	0.724
U-Net	100%	OBP+OIC	0.98	0.775	0.837	0.724

PT = Prediction Threshold, Dice = Dice's coefficient, FS = fixed-stride, OBP = object-based positioning, OBP+OIC = object-based positioning + object integrity constraint
