# Supplementary material for: Improving deep learning-based segmentation of diatoms in gigapixel-sized virtual slides by object-based tile positioning and object integrity constraint
Source: PLoS One. 2023 Feb 24;18(2):e0272103. doi: 10.1371/journal.pone.0272103 (PMC9956069; doi:10.1371/journal.pone.0272103)
Supplement: Attachment — Submitted filename: Response to the Editor.docx [file pone.0272103.s011.docx]

PONE-D-22-19514R2

Improving deep learning-based segmentation of diatoms in gigapixel-sized virtual slides by object-based tile positioning and object integrity constraint Dr. Michael Kloster

Dear Dr. Kloster,

We've checked your submission and before we can proceed, we need you to address the following issues:

1. Thank you for stating the following financial disclosure:

"This work was funded by the Deutsche Forschungsgemeinschaft (DFG) in the framework of the priority programme SPP 1991 Taxon-OMICS under grant nrs. BE4316/7-1 & NA 731/9-1. D.L.'s contribution was supported by the German Federal Ministry for Economic Affairs and Energy (BMWi) (FKZ: 0324254D). BIIGLE is supported by the BMBF-funded de.NBI Cloud within the German Network for Bioinformatics Infrastructure (de.NBI) (031A537B, 031A533A, 031A538A, 031A533B, 031A535A, 031A537C, 031A534A, 031A532B)."

Please state what role the funders took in the study. If the funders had no role, please state: "The funders had no role in study design, data collection and analysis, decision to publish, or preparation of the manuscript."

The funders had no role in study design, data collection and analysis, decision to publish, or preparation of the manuscript.

If this statement is not correct you must amend it as needed.

Please include this amended Role of Funder statement in your cover letter; we will change the online submission form on your behalf.

The statement is included in the cover letter now.

2. Please ensure that you refer/cite to Table 1-4 in your text as, if accepted, production will need this reference to link the reader to the Table.

All table, figures and supplemental information are referenced using the citation system integrated in MS-WORD. A (minor) problem we have with this approach is that we did not find a way to address multiple figures in the way described in the authoring guidelines (e.g. Figs 3 and 4, Figs 5-7) and that the bold font formatting from the figure / table caption is also used on the reference links.

Larger files of the supplemental information are available via Zenodo, but we did not upload some figures / tables yet to any repository and thus included them in the manuscript. Please drop us a note if we are supposed to do so, from my understanding there will be uploaded to FigShare by PLOS ONE.
